# Supplementary material for: Expanding Pharmacotherapy Data Collection, Analysis, and Implementation in ERAS® Programs—The Methodology of an Exploratory Feasibility Study
Source: Healthcare (Basel). 2020 Aug 3;8(3):252. doi: 10.3390/healthcare8030252 (PMC7551795; doi:10.3390/healthcare8030252)
Supplement: Supplementary file 1 [file healthcare-08-00252-s001.pdf]

Enhanced Recovery Comparative Pharmacotherapy Collaborative (ERCPC) Data Dictionary of Pharmacotherapy Elements

Since 2012, the Enhanced Recovery After Surgery (ERAS®) Society has published guidelines pertaining to perioperative care in adult elective colorectal and gynecologic/oncology surgery patients. These bundled guidelines contain recommendations on the use of pharmacologic therapy, including prophylaxis against (1) surgical site infection (SSI), (2) thromboembolism (VTE), and (3) postoperative nausea and vomiting (PONV), among others. While the guidelines provide high-quality evidence on the use of pharmacotherapy in each of these ERAS® elements, the specifics about agent selection and dosing regimens are limited. These dosing variables include: (a) medication administration time in relation to the procedure; (b) name and dose of medication used; and (c) duration of therapy in and out of the hospital. Literature suggests that lack of effective prophylaxis to address SSI, VTE, and PONV is associated with significant clinical morbidity, and these adverse outcomes may be independent drivers of hospital length of stay (LOS). Suboptimal preventive pharmacotherapy may lead to increased frequency and severity of complications as well as delayed patient discharge to a home setting.

This exploratory feasibility study will attempt to characterize the optimal use of pharmacotherapy indicated for prophylaxis against **surgical site infection**, **venous thromboembolism**, and **post-operative nausea and vomiting** in these surgical patients. Further, it will generate hypotheses that can be tested using a multi-site, multi-procedural methodology within Enhanced Recovery After Surgery Interactive Audit System (EIAS)® related to these three primary outcome measures. Data collection will be facilitated through REDCap mobile.

I. From EIAS®—Including Patient Demographic Information (Gender, Age, Weight, eGRF)

Complications: the recording of a deviation from the normal post-operative course event in EIAS® (Clavien-Dindo scaling). One space is needed for entry [1-5].

Length of stay (LOS): the recording of the number of days of residence in the hospital (whole number). Two spaces are needed for entry.

II. Data Elements and Space Requirements for Data Collection from Healthcare Records Include

CPT code for procedure: the primary Current Procedural Terminology for the operative procedure as listed on the face sheet. Five spaces are needed for the entry.

ICD code for diagnoses: the diagnostic codes associated with the patient. Six spaces are needed for the entry for up to 3 codes.

Pharmacologic Agent: the active pharmaceutical ingredient (API) specified in the medication order. All APIs will be listed in a drop-down menu.

Dose: the amount of each medication administered to the patient as specified in the medication order expressed in mg, grams, or units and recorded on the Medication Administration Record (MAR). Doses will be entered numerically. Five spaces are needed for entry.

Proximity of dose administration to the incision: the amount of time in minutes between the administration time of a medication and the surgical incision time as specified in the medication order and recorded on the MAR. Three spaces will be needed for entry.

Duration of therapy: the number of doses administered to the patient during the hospital stay (PREOP, INTRAOP, and POSTOP) as recorded on the MAR and/or at home. Three data fields with two spaces are needed for entry.

Patient stratification of risk scoring used: evidence of use of any scoring system related patient risk assessment to the need for prophylaxis. This is a dichotomous variable (Y/N).

III. REDCap Data Collection Template

A. Patient demographics (numerical entry)

1. Age
2. Gender
3. Weight (in kg)
4. eGFR

B. Current Procedural Terminology code (dropdown menu)

5. Primary

C. ICD diagnostic code(s) (dropdown menu)

6. First
7. Second
8. Third

D. Complications (Grades I–V) (up to 4 complication levels) (dropdown menu)

9. Grade a
10. Grade b
11. Grade c
12. Grade d

E. Length of stay (numerical entry)

13. Days

F. Antimicrobial SSI prophylaxis and skin preparation

Pharmacologic Agents (up to 4 antibiotics) (dropdown menu)

ampicillin/sulbactam
cefazolin
cefotetan
cefoxitin
ceftriaxone
ciprofloxacin
ertapenem
gentamicin
levofloxacin
meropenem
metronidazole
piperacillin/tazobactam
tobramycin
other (type-in)

14. Antibiotic a
15. Antibiotic b
16. Antibiotic c
17. Antibiotic d
18. Primary Antibiotic Dose (in grams) (numerical entry)

Weight-adjusted (radio button)

19. Yes
20. No

Skin preparation (radio button)

21. povidone-iodine
22. chlorhexidine
23. none
24. *Proximity of administration to incision (in minutes) (numerical entry)*

Duration of therapy (in number of doses) (numerical entry)

25. PRE-OP
26. INTRA-OP (REDOSING NEEDED?)
27. POST-OP

Patient stratification of risk for infection scoring used (radio button)

28. Yes
29. No

Surgical site infection (dropdown menu)

30. None
31. Superficial
32. Deep
33. Organ space

G. Prophylaxis against venous thromboembolism (VTE)

Pharmacologic Agents (up to 2 agents) (dropdown menu)

apixaban (mg)
dalteparin (units)
enoxaparin (mg)
rivaroxaban (mg)
tinzaparin (units)
unfractionated heparin (units)
other (type-in)

34. Agent a
35. Agent b

Dose (in mg or units) (numerical entry)

36. Agent a
37. Agent b

Duration (in doses) (numerical entry)

38. In hospital
    a. PRE-OP
    b. POST-OP in hospital
39. Post discharge

Weight adjusted (radio button)

40. Yes
41. No

Renal function adjusted (radio button)

42. Yes
43. No

Proximity of first dose administration to incision (in minutes) (numerical entry)

44. Agent a
45. Agent b

Patient stratification of risk of VTE scoring used (radio button)

46. Yes
47. No

H. Post-Operative Nausea and Vomiting (PONV) Prophylaxis

Pharmacologic Agents (up to 4 agent given pre-operatively) (dropdown menu)

aprepitant
dexamethasone
droperidol
haloperidol
granisetron

metoclopramide
ondansetron
prochlorperazine
promethazine
scopolamine
other (type-in)

48. Agent a
49. Agent b
50. Agent c
51. Agent d

Doses (in mg) (numerical entry)

52. Agent a
53. Agent b
54. Agent c
55. Agent d

Proximity to incision (in minutes) (numerical entry)

56. Agent a
57. Agent b
58. Agent c
59. Agent d

Duration of therapy (for any anti-emetic) (numerical entry)

71. PRE-OP
72. POST-OP

Patient stratification of risk of PONV scoring used (radio button)

73. *Yes*
74. *No*
